# Evaluation of the use of sonication combined with enzymatic treatment for biofilm removal in the microbiological diagnosis of prosthetic joint infection

**DOI:** 10.1128/spectrum.00020-24

**Published:** 2024-06-25

**Authors:** Lucía Henríquez, Carmen Martín, Maite Echeverz, Íñigo Lasa, Carmen Ezpeleta, María Eugenia Portillo

**Affiliations:** 1 Department of Clinical Microbiology, University Hospital of Navarra, Institute of Healthcare Research of Navarra (IdiSNa), Pamplona, Spain; 2 Laboratory of Microbial Pathogenesis, Navarrabiomed, Public University of Navarra (UPNA), Institute of Healthcare Research of Navarra (IdiSNa), Pamplona, Spain; 3 CIBER, Epidemiología y Salud Pública, (CIBERESP), Madrid, Spain; Instituto de Investigacion Sanitaria Gregorio Maranon, Madrid, Spain

**Keywords:** biofilm disruption, enzymatic treatment, prosthetic joint infection, sonication

## Abstract

Sonicating explanted prosthetic implants to physically remove biofilms is a recognized method for improving the microbiological diagnosis of prosthetic joint infection (PJI); however, chemical and enzymatic treatments have been investigated as alternative biofilm removal methods. We compared the biofilm dislodging efficacy of sonication followed by the addition of enzyme cocktails with different activity spectra in the diagnosis of PJI with that of the sonication of fluid cultures alone. Consecutive patients who underwent prosthesis explantation due to infection at our institution were prospectively enrolled for 1 year. The diagnostic procedure included the collection of five intraoperative tissue cultures, sonication of the removed devices, and conventional culture of the sonication fluid. The resulting sonication fluid was also treated with an enzyme cocktail consisting of homemade dispersin B (0.04 µg/mL) and proteinase K (Sigma; 100 µg/mL) for 45 minutes at 37°C. The resulting sonication (S) and sonication with subsequent enzymatic treatment (SE) fluids were plated for aerobic and anaerobic culture broth for 7 days (aerobic) or 14 days (anaerobic). Identification was performed by matrix-assisted laser desorption ionization–time-of-flight mass spectrometry (Bruker). We included 107 patients from whom a prosthetic implant had been removed, among which PJI was diagnosed in 36 (34%). The sensitivity of S alone was significantly greater than that of SE alone (82% vs 71%; *P* < 0.05). Four patients with PJI were positive after sonication alone but negative after sonication plus enzymatic treatment. The four microorganisms missed after the addition of the enzyme cocktail were *Staphylococcus aureus*, two coagulase-negative *Staphylococci,* and *Cutibacterium acnes*. In conclusion, sonication alone was more sensitive than sonication followed by enzymatic treatment. The combination of these two methods had no synergistic effect; in contrast, the results suggest that the combination of both dislodgment methods affects the viability of gram-positive microorganisms.

**IMPORTANCE:**

While the potential of sonication and enzymes as biofilm dispersal agents has been previously described, the originality of our work resides in the combination of both methods, which is hypothesized to enhance the ability to remove biofilm and, therefore, improve the microbiological diagnosis of PJI

## INTRODUCTION

The pathogenesis of prosthetic joint infection (PJI) is a result of the associated biofilms, which make these infections difficult to dislodge and identify using traditional microbiological techniques. *Staphylococcus aureus* and coagulase-negative *Staphylococci* are frequently identified as the primary pathogens responsible for PJI (1). Microorganisms in biofilms are less metabolically active because they are embedded in an extracellular matrix and adhere to the implant surface (2). Accurate diagnosis of PJI is critical for successful treatment. Therefore, sonicating removed implants has been shown to improve culture sensitivity for PJI identification by physically removing biofilms (3–7). However, some PJIs remain culture-negative (CN), which can be partly explained by insufficient biofilm removal (8, 9). Consequently, the need for new biofilm disruption methods or adjuvant treatments to facilitate biofilm removal has been recognized and documented in the literature (10).

The addition of chemical agents such as dithiothreitol (DTT) or the chelating agent ethylenediaminetetraacetic acid has recently been proposed as an inexpensive alternative to sonication (11). However, it remains unclear whether inoculation with chemical agents negatively affects the sensitivity of cultures (12).

Moreover, the use of hydrolytic enzymes capable of degrading matrix components has been investigated as a strategy to combat biofilm-associated infections (13). The aim of earlier research was to use enzymes to disrupt biofilms formed by both methicillin-resistant *S. aureus* (MRSA) and methicillin-sensitive *S. aureus*. Nevertheless, a significant obstacle impeding the progress of enzymatic antibiofilm treatments is the intricate composition of the extracellular polymeric substance matrix (14)

Enzymes such as glycosidases and proteases have the potential to degrade the extracellular matrix (15), resulting in the release of planktonic cells and their constituents, which can be more easily accessed by antibiotics (16). Polysaccharides such as poly-N-acetylglucosamine (PNAG) play a key role in biofilm formation. The *ica*ADBC locus, which is responsible for the synthesis of PNAG, is present in the majority of staphylococcal isolates from implant-associated infections (17).

Dispersin B is an endoglycosidase that can hydrolyze PNAG (13). As proteins are major components of biofilm matrixes, proteases are considered potential enzymes for biofilm removal. Proteinase K, known for its stability under various conditions, including pH, buffer salts, detergents, and temperatures, contributes to the degradation process for biofilm detachment (10). Therefore, the role of enzymes has been mainly investigated in terms of the prevention of biofilm formation, especially *in vitro* biofilms (18, 19).

The synergistic effects of combining enzymes with antibiotics (13) or antiseptics (20), both of which are chemical methods, against biofilms have been evaluated in previous studies. Conversely, the combination of a physical method, such as sonication, and enzymatic treatment has not been evaluated. Previous studies have outlined the potential of using sonication and enzymes as distinct methods for biofilm disruption (17–23). The objective of this study was to investigate whether hydrolytic enzymes could augment the biofilm removal efficacy of sonication, a physical disruption method, specifically for the diagnosis of PJI. To our knowledge, this study represents the first instance in which a combination of removal techniques involving conventional sonication fluid and enzymatic treatment has been conducted.

## MATERIALS AND METHODS

### Study design

A prospective multicentric cohort study was conducted in four tertiary medical care centers, Hospital Universitario de Navarra (≈1,000 beds), Hospital Reina Sofia de Tudela (≈200 beds), Hospital García Orcoyen de Estella (≈100 beds), and Clínica Ubarmin, covering the entire population of Navarra (≈700,000 inhabitants), in northern Spain.

### Study population

We prospectively included all consecutive patients aged ≥18 years from the participating hospitals who were hospitalized for 1 year—from January to December 2017—in whom a joint prosthesis was removed for any reason. The following information was recorded: demographic, clinical, radiological, laboratory, and microbiological data and information on the type of surgical management and antimicrobial therapy. A long-term follow-up of 5 years was performed via biannual follow-up visits.

### Study definitions

PJI was defined according to the European Bone and Joint Infection Society definition criteria (21). Aseptic failure (AF) was determined when the prosthesis was removed in the absence of the PJI criteria. Previous antimicrobial treatment was defined as the administration of any antimicrobial agent for at least 1 day within the 14 days prior to surgery. Preoperative prophylaxis was given to all patients.

### Periprosthetic tissue samples

Tissue samples were intraoperatively collected in native vials. Aliquots of tissue specimens were inoculated in Schaedler enriched with 5% sheep blood agar (BioMérieux, Marcy L’Etoile, France), PolyViteX agar (BioMérieux, Marcy L’Etoile, France), and thioglycollate broth (BBL Enriched Thioglycollate Medium with Vitamin K and Hemin; Beckton Dickinson and Company, USA). Aerobic cultures were incubated at 37°C for 1 week, and anaerobic cultures were incubated for 2 weeks.

### Sonication of the removed implants

The removed orthopedic prostheses were aseptically explanted in the operating room and transported to the microbiology laboratory in solid polyethylene containers with screw caps and airtight internal seals. The removed prostheses were sonicated in the microbiology laboratory (sonicator model SM25E-MT, Branson Ultrasonics Corporation, Geneva, Switzerland) for 1 minute at a frequency of 40 ± 5 kHz as previously described (24).

### Enzymatic treatment

The resulting sonication fluid was treated with an enzyme cocktail consisting of homemade dispersin B (0.04 µg/mL) and proteinase K (Sigma; 100 µg/mL) for 45 minutes at 37°C.

### Bacterial viability after enzymatic treatment

**Fig 1:**
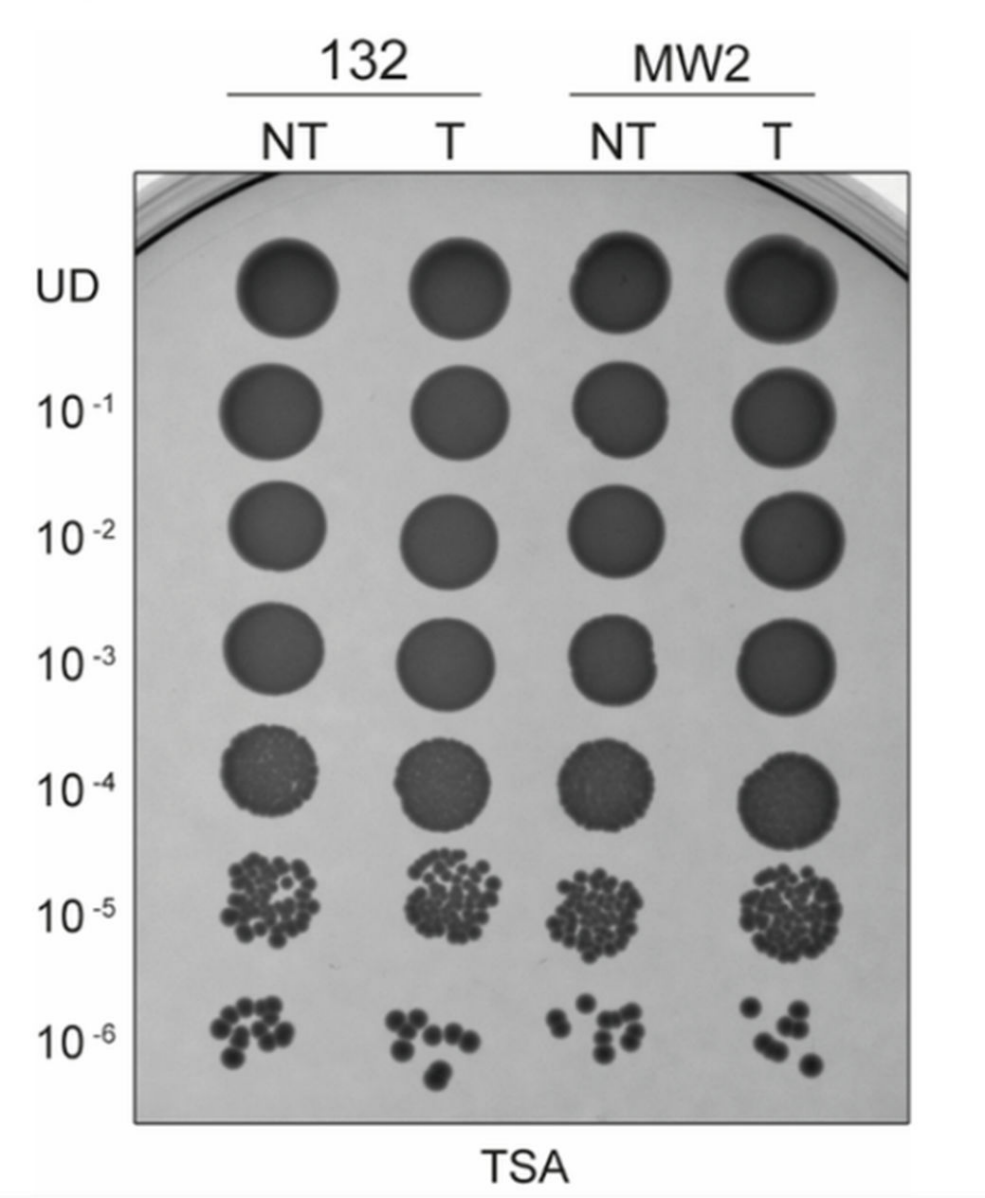
Viability of *S. aureus* strains 132 and MW2 after treatment with serial dilutions of proteinase K (100 µg/mL) and dispersin B (0.04 µg/mL) for 45 minutes at 37°C. NT, T, and UD in a TSA plate.

The viability of clinical *S. aureus* strains 132 and MW2 was determined after treatment with proteinase K (100 µg/mL) and dispersin B (0.04 µg/mL) for 45 minutes at 37°C. Five-microliter portions of the appropriate dilutions of each culture were incubated for 24 hours at 37°C on a trypto-casein soy agar (TSA) plate, and the following types of samples were included: nontreated (NT), treated (T), and undiluted culture (UD). The clinical strains used were *S. aureus* MW2 (a typical community-acquired MRSA strain that was isolated in 1998 in North Dakota, USA) (22) and *S. aureus* 132 (a clinical MRSA strain that is bioﬁlm-positive) (23). The bacterial viability study is outlined in Fig. 1.

### Sonication fluid and sonication with subsequent enzymatic treatment fluid culture

**Fig 2:**
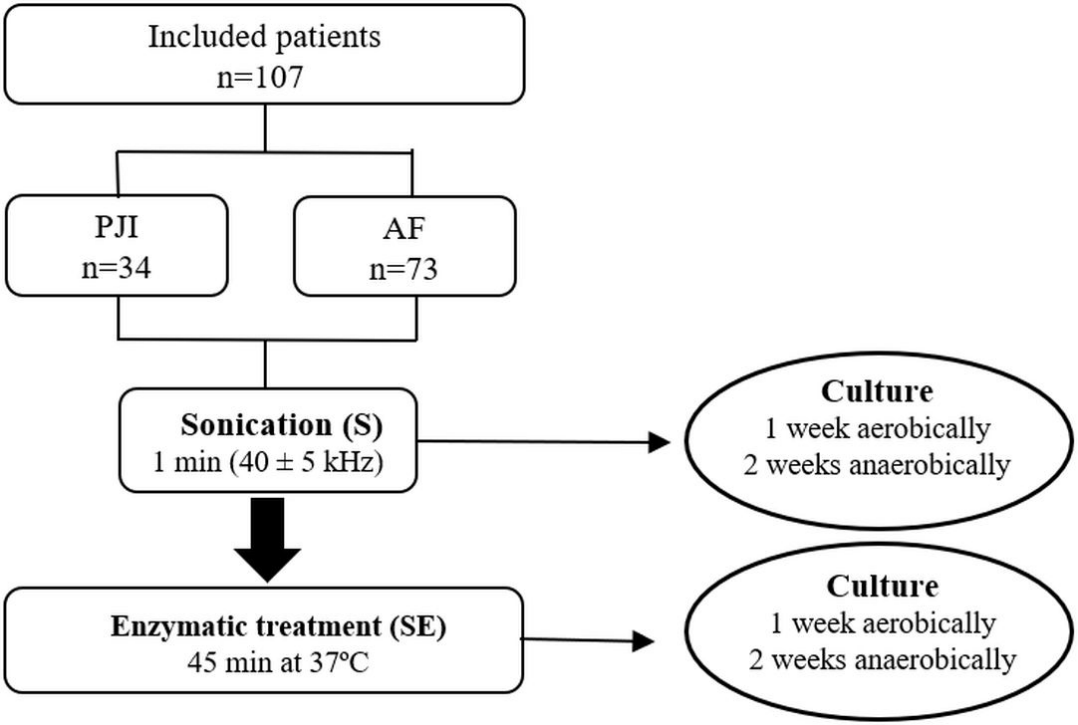
Biofilm disruption study workflow. Sonication (S) vs sonication with subsequent enzymatic treatment (SE).

Sonication (S) and sonication with subsequent enzymatic treatment (SE) fluids were acquired from all samples (including those from the PJI and AF patients). One-milliliter aliquots of each of the resulting S and SE were plated on Schaedler agar plates enriched with 5% sheep blood (BioMérieux, Marcy L’Etoile, France), PolyViteX agar plates (BioMérieux, Marcy L’Etoile, France), and thioglycollate broth (BBL Enriched Thioglycollate Medium with Vitamin K and Hemin, Beckton Dickinson and Company, USA). Cultures were incubated aerobically at 37°C for 1 week or anaerobically for 2 weeks. S and SE cultures were considered positive if ≥50 colony-forming units (CFUs) per milliliter of an organism with the same morphology grew, as previously defined (24). If the patient had previously received an antimicrobial agent, a sonication fluid culture was considered positive with growth of ≥1 CFU/mL. Microorganisms grown on culture plates were also subjected to matrix-assisted laser desorption ionization–time-of-flight mass spectrometry (Bruker) analysis according to the manufacturer’s instructions. The study design is summarized in Fig. 2.

### Statistical analysis

Comparisons between categorical variables were performed using McNemar’s χ^2^ test or Fisher’s exact test, as appropriate. Continuous variables were compared using the Mann‒Whitney *U* test. Probability (*P*) values less than 0.05 were considered to indicate statistical significance. Calculations were performed and graphs were generated using Prism software (version 10.1; GraphPad, La Jolla, CA).

## RESULTS

### Study population

We included 107 patients from whom a prosthetic implant had been removed. AF was diagnosed in 73 patients (68%), and PJI was diagnosed in 34 patients (32%). Twenty-four of the patients with PJI (71%) received antimicrobial treatment within 14 days prior to sampling. Additional characteristics of the 73 patients are summarized in Table 1.

**TABLE 1:** Characteristics of the 107 patients in the study who had aseptic failure and a prosthetic joint infection^*a*^

	Aseptic failure (*n* = 73)	Prosthetic joint infection (*n* = 34)	*P*-value
Median patient age in years (range)	70 (35–86)	73 (48–87)	
Male sex	43 (59%)	15 (43%)	0.1529
Type of prosthesis			
Knee (*n* = 45)	41 (56%)	23 (68%)	0.157
Hip (*n* = 30)	30 (41%)	9 (26%)	0.01054
Shoulder (*n* = 1)	2 (3%)	1 (3%)	0.9531
Ankle (*n* = 1)	0	1 (3%)	0.141
Type of infection			
Early postoperative infection (<3 months after surgery)	0	5 (15%)	
Delayed infection (3–24 months after surgery)	0	22 (65%)	
Late infection (>24 months after surgery)	0	7 (21%)	
No. of patients who received antibiotics prior to sampling	0	24 (71%)	

^*a*^ Values represent numbers (%) if not indicated otherwise.

### Comparison of biofilm disruption methods

Table 2 summarizes the accuracy of using traditional sonication and sonication combined with enzymatic treatment methods for biofilm disruption. The sensitivity of S (82%) was significantly greater than that of SE (71%; *P* < 0.05).

**TABLE 2:** Diagnostic performance of two biofilm disruption methods for the 107 patients from which implants were removed (34 with prosthetic joint infection and 73 with aseptic failure)^*a*^^,*b*^

Diagnostic method	Sensitivity (%) (95% CI)	Specificity (%) (95% CI)	PPV (%) (95% CI)	NPV (%) (95% CI)
Conventional sonication fluid	82 (66–92)	100 (95–100)	100 (88–100)	92 (84–96)
Sonication + enzymatic treatment	71 (54–83)	100 (95–100)	100 (86–100)	88 (79–93)

^*a*^ 95% CI, 95% confidence interval; PPV, positive predictive value; NPV, negative predictive value.

^*b*^ The cultures of 73 implants removed due to aseptic failure were negative after both disruptive methods were performed.

### Microbiological findings

All S and SE cultures from AF patients were negative. Table 3 summarizes the microbiological findings of each diagnostic method for detecting biofilm disruption from the 34 patients with PJI. In 24 cases, the same microorganisms were detected by both methods. More pathogens were detected in the conventional S cultures than in the SE cultures (28 vs 24 microorganisms, respectively). There were two cases of PJI in which no growth was detected by either method (S or SE), but *S. aureus* and *Candida parapsilosis* were isolated from periprosthetic tissue cultures. The detection of polymicrobial infections (i.e., the isolation of ≥2 microorganisms) was not affected by the combination of disruption methods. Negative cultures were observed less frequently with conventional S than when the combination of both methods was applied (18% vs 29%, *P* < 0.01).

**TABLE 3:** Microbiological findings from 34 prosthetic joint infections stratified by the type of biofilm disruption diagnostic method

Characteristic	Conventional sonication fluid culture	Sonication fluid combined with enzymatic treatment
No. of microorganisms detected		
0	6 (18%)	10 (29%)
1	26 (76%)	22 (65%)
≥2	2 (6%)^*a*^	2 (6%)^*a*^
Total no. of microorganisms isolated	28	24
Gram-positive cocci	22 (73%)	19 (73%)
Coagulase-negative *Staphylococci*	16	14
*S. aureus*	5	4
*Enterococcus* spp.	1	1
Gram-negative bacilli	3 (10%)	3 (12%)
*Escherichia* spp.	2	2
*Enterobacter* spp.	1	1
Anaerobe	4 (13%)	3 (12%)
*C. acnes*	4	3
Other microorganisms	1 (3%)	1 (4%)
*Corynebacterium* spp.	1	1

^*a*^ *Staphylococcus epidermidis* + *Staphylococcus caprae*; *Enterobacter cloacae* + *Escherichia hermannii*.

### Effect of previous antimicrobial treatment on the quantification of microorganisms after performing both biofilm disruption methods

**Fig 3:**
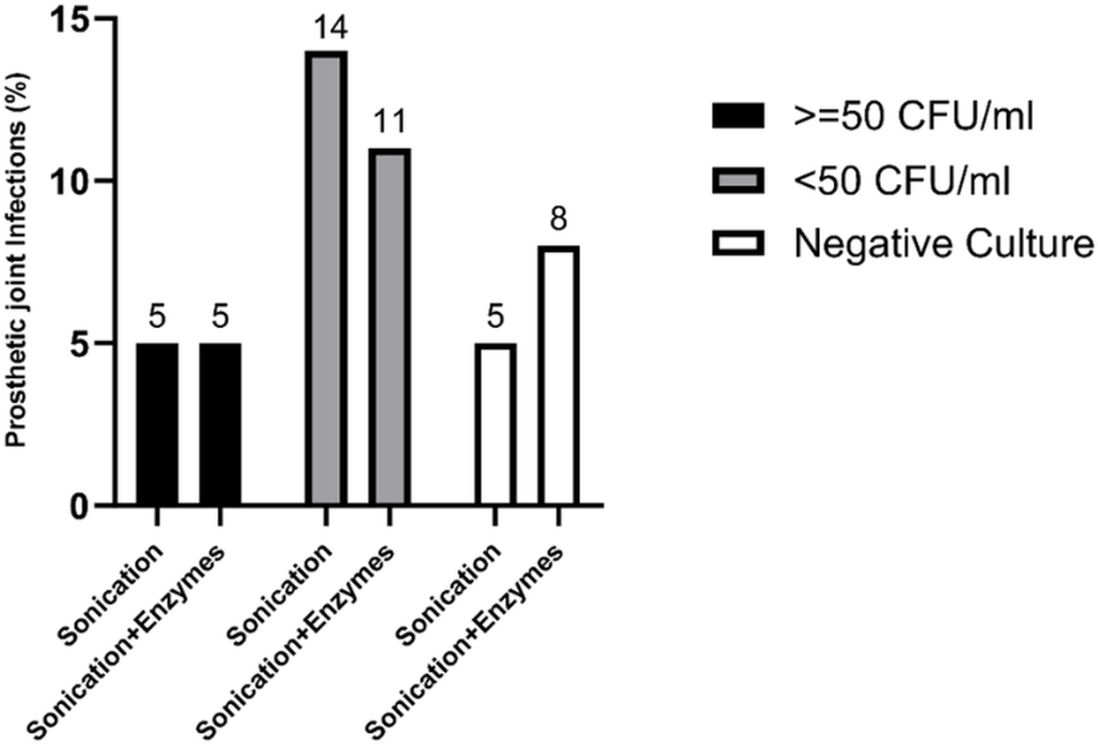
Effect of previous antimicrobial treatment on the quantification of microorganisms after both biofilm disruption methods.

Figure 3 illustrates the percentage of PJIs with an etiological diagnosis based on conventional S and SE cultures in patients who had undergone antibiotic treatment within 14 days before surgery. Sonication enabled the isolation of three additional pathogens with counts ≤50 CFU/mL from patients receiving antibiotics. The two PJIs that did not exhibit growth in the S and SE cultures but were isolated in periprosthetic tissue cultures were identified in patients who had previously received antimicrobial treatment. Of the microorganisms with growth below the cutoff of ≤50 CFU/mL, 86% were from patients who had undergone antimicrobial treatment after both disruption methods. Furthermore, among patients receiving antibiotic treatment prior to sample collection, three negative SE cultures displaying growth <50 CFU were isolated with conventional S culture. These three infections were categorized as hematogenous late infections (>3 years).

## DISCUSSION

Although the use of sonication improves the diagnosis of PJI, a significant number of these infections are CN (9). CN PJI may be a result of prior antimicrobial use or inadequate diagnostic methods, such as insufficient biofilm removal from the implant or excessive ultrasound power and/or exposure time (25).

Various approaches to biofilm removal have been investigated in recent years. Various chemical agents have been assessed for their ability to remove biofilms *in vitro* (26), and subsequently, several authors have evaluated the performance of the commercial diagnostic kit MicroDTTect for the diagnosis of orthopedic infections (27). Randau et al. reported that sonication was more sensitive (75% vs 65%) but less specific (85% vs 10%) than the DTT system for the diagnosis of PJI (28). This group reported that the low pH of the chemical fluid in the DTT system was associated with false-negative results. Karbysheva et al. showed that the MicroDTTect system had a significantly lower sensitivity than sonication (43% vs 74%, *P* < 0.05) but comparable specificity (29). The concentration of the chemical agent used seems to be critical. Some authors claim that when DTT is used at a concentration of 0.1% or 1 g/L, it has no effect on bacterial viability (30).

Enzymes have replaced toxic chemicals or reduced their use due to the various limitations of the chemicals used for biofilm removal (16). Enzymes such as glycosidases, proteases, and DNases have the potential to degrade the extracellular matrix, resulting in the release of planktonic cells and their components, which are easier to isolate (15).

Although the potential of using sonication and enzymes for biofilm disruption has been previously described in the literature (17–26), the novelty of our study lies in the integration of these methods. This combination has been hypothesized to enhance the biofilm removal capacity, consequently improving the microbiological diagnosis of PJI. Previous investigations have revealed that sonicating enzymes can cause them to unfold and subsequently become inactive, primarily due to their protein nature (31, 32). This poses a limitation when investigating the synergy of both procedures in biofilm disruption. Therefore, in our analysis, the enzyme cocktail was introduced after the sonication of the explanted prosthesis to address this limitation. Unexpectedly, when the enzyme cocktail was added to the sonication fluid, the sensitivity of the cultures was significantly lower than that of the conventional sonication fluid (71% vs 82%; *P* < 0.05). The combination of both disruption methods did not reduce the rate of CN PJI but increased it from 18% (sonication alone) to 29%. Interestingly, all but one of the six patients with negative sonication fluid cultures received antibiotics within the 14 days prior to surgery, as all were acute infections (one early infection and five late infections). All of the late infections occurred more than 2 years after surgery, so they were presumably hematogenous infections, but unfortunately, blood samples were not taken from any of them. These findings are consistent with previous results suggesting that biofilms in acute PJI are immature and sonication does not show good biofilm removal efficacy in acute PJI (33). In addition, other studies have shown that sonication does not improve diagnostic sensitivity compared to other less aggressive techniques, such as vortexing, for acute infections due to the immature nature of the biofilm (24). This is consistent with our data, in which the CN infections were mostly acute, and suggests that increasing the aggressiveness of treatment, such as by combining a physical with an enzymatic disruptive method, in infections where the biofilm is not mature may have resulted in a loss of bacterial viability. We suggest that the combination of both treatments affects bacterial viability since, prior to this study, the viability of the *S. aureus* strains was tested via enzymatic treatment.

On the other hand, antibiotic treatment is administered early in acute infections, which negatively affects the recovery of pathogens in culture. In these situations, it is unclear whether CN PJIs are a consequence of the diagnostic technique used or due to the absence of microbial growth as a result of antibiotic exposure prior to sampling (34). The only patient who did not receive antibiotics prior to sampling was diagnosed with PJI caused by *C. parapsilosis* growing in two periprosthetic tissues. Curiously, the addition of enzymes after the sonication procedure had no synergistic effect. On the other hand, 4 of the 28 microorganisms isolated by sonication (14%) were lost by the addition of enzymes. Of the four infections that were detected by S alone, four were acute infections caused by coagulase-negative *Staphylococci* (*n* = 2) and *S. aureus* (*n* = 1), and one was a late infection caused by *Cutibacterium acnes* (*n* = 1). The missed pathogens were all gram-positive microorganisms, which are good targets for the enzyme cocktail used. Indeed, it has been shown that treating biofilms with protease (10 µg/mL) alone does not affect bacterial viability (16). These results suggest that this combination of methods may be detrimental to the microorganisms, confirming the superior efficacy of physical removal alone.

Depending on the frequency, intensity, and energy applied during sonication, subcellular structures or protein complexes can be disrupted, and proteins can be denatured due to the heat generated. To avoid these problems, the enzyme cocktail was applied after rather than during sonication. In addition, there is evidence that sonication induces the formation of aggregates that may cause toxicity (31, 32).

An alternative approach could involve conducting enzyme treatment before sonication. However, we were unable to adopt this method due to our hospital’s medical protocol for diagnosing implant-associated infection, which mandates sonication of the implant followed by culture of the sonication fluid. Therefore, the sonication fluid was first collected for further treatment with the enzyme cocktail. Another limitation of our study is that we did not investigate the different biomaterials that had been used to produce the prostheses, which could influence adhesion and biofilm formation. Furthermore, we did not measure the pH of the fluids, which prevented us from establishing a correlation with the observed lack of synergy. Notably, the strengths of our study include the use of *in vivo* biofilms and a significantly longer follow-up period, which ensures a robust diagnosis of infection. In conclusion, sonication combined with an enzyme cocktail consisting of proteinase K and dispersin B did not reduce the rate of PJI in negative cultures. Sonication alone showed higher sensitivity and specificity in the diagnosis of PJI, especially in patients who had received antibiotics prior to surgery.
